# Hypoxia Induces Apoptosis of Microglia BV2 by Upregulating Kir2.1 to Activate Mitochondrial-Related Apoptotic Pathways

**DOI:** 10.1155/2022/5855889

**Published:** 2022-03-17

**Authors:** Yu-fang Xie, Yan Wang, Yi Rong, Wenjun He, Meijuan Yan, Xinzhi Li, Junqiang Si, Li Li, Yingying Zhang, Ketao Ma

**Affiliations:** ^1^Key Laboratory of Xinjiang Endemic and Ethnic Diseases, Ministry of Education, Shihezi University School of Medicine, Shihezi, China; ^2^NHC Key Laboratory of Prevention and Treatment of Central Asia High Incidence Diseases, First Affiliated Hospital, Shihezi University School of Medicine, Shihezi, China; ^3^Department of Physiology, Shihezi University School of Medicine, Shihezi, China; ^4^Department of Pathophysiology, Shihezi University School of Medicine, Shihezi, China; ^5^Department of Physiology, Medical College of Jiaxing University, Jiaxing, Zhejiang, China; ^6^Clinical Laboratory of the First Affiliated Hospital, Shihezi University School of Medicine, Shihezi, China

## Abstract

**Aim:**

To explore the role of Kir2.1 in hypoxia-induced microglial apoptosis.

**Methods:**

BV2 microglial cell lines were cultured and treated with ML133 hydrochloride, a Kir2.1 channel blocker, for 23 h and with 500 *μ*mol/L of CoCl_2_ for 8 h. Cells were divided into the control, CoCl_2_ (hypoxia-induced model), and CoCl_2_+ML133 (hypoxia-induced model established after ML133 pretreatment) groups. Cell activity was assessed using the CCK-8 technique. The membrane potential and Kir2.1 current of BV2 were evaluated with the whole-cell patch-clamp technique. The protein levels and mRNA levels of Kir2.1, apoptotic proteins Bax and caspase-3, and antiapoptotic protein Bcl-2 in BV2 cells were evaluated via immunofluorescence, Western blot analysis, and real-time quantitative reverse transcription. The apoptosis rate of BV2 cells was detected via flow cytometry.

**Results:**

CCK-8 analysis showed that the cell activity of each group increased initially and then decreased. The 2 h intervention group had the highest cell activity, and that of the 8 h group was >90%. Hence, there was a significant difference in the results (*P* < 0.05). Western blot analysis revealed that the expression of cleaved caspase-3 significantly increased in the 8 h group compared with the 0 h group. Compared with the control group, the expression of Kir2.1 and mRNA in the CoCl_2_ group increased. Thus, hypoxia could upregulate the expression of Kir2.1. The whole-cell patch-clamp results showed that the Kir2.1 channel current amplitude of the CoCl_2_ group increased compared with that of the control group. Therefore, hypoxia could enhance Kir2.1 function. The apoptosis rate of the CoCl_2_ group was significantly higher than that of the control group. Further, the ML133 group had a significantly lower apoptosis rate than the CoCl_2_ group. The expression of apoptotic proteins Bax and cleaved caspase-3 increased in the CoCl_2_ group, and that of the antiapoptotic protein Bcl-2 decreased. The expression of apoptotic proteins Bax and cleaved caspase-3 reduced in the CoCl_2_+ML133 group, whereas that of the antiapoptotic protein Bcl-2 increased.

**Conclusion:**

Hypoxia can induce microglia BV2 apoptosis accompanied by the upregulation of Kir2.1 and mRNA expression levels and an increase in the Kir2.1 current. Moreover, ML133 can inhibit hypoxia-induced BV2 cell apoptosis. Hence, Kir2.1 may be involved in the process of hypoxia-induced BV2 cell apoptosis.

## 1. Introduction

Trigeminal neuralgia (TN) occurs in the distribution of one or more branches of the trigeminal nerve; it is commonly characterized by unilateral, severe, temporary, stabbing, and recurrent pain, which affects the quality of life of patients [1, 2]. Microglia are the primary neuroimmune cells that maintain homeostasis, and they play a surveillance and defense role. These cells are the primary sensors of pathophysiological processes, triggering cascade reactions [3]. Moreover, they play an important role in oral and facial pain such as that in TN [4–6]. Microglia and astrocytes, which are overactive in the trigeminal sensory nucleus, are involved in the development and maintenance of neural behavior if nerve injury occurs. The activation of glia is essential in the development and persistence of neuropathic pain [7]. Oxygen homeostasis is critical to the health of mammals [8]. Previous studies have found that hypoxia can induce oxidative stress in cells [9]. Interestingly, there was a significant increase in the expression of oxidative stress byproducts released by immune cells clustered at the site of nerve injury, which may be a mechanism that induces trigeminal pain [10]. CoCl_2_ can successfully induce cardiac myocyte H9C2 hypoxia [11]. Reactive oxygen species (ROS), an oxidative stress product produced by hypoxia, can cause irreversible damage to the cell membranes, DNA, and other cell structures by oxidizing lipids, proteins, and nucleic acids, which then leads to cell apoptosis [12].

Potassium ion channels play an important role in regulating the development of apoptosis and can delay or even inhibit apoptosis. Kir2.1 is a subtype of an inward rectifier potassium channel, which has important physiological functions. These include the regulation of potassium ion transduction and maintenance of intracellular potassium ion concentration in a relatively stable state as well as the control of the cellular electrical signal transmission of cell membrane potential and nerves, the secretion and release of hormones such as insulin, and cell metabolism and maintenance of vascular tension. The Kir2.1 channel is essential in maintaining the normal function of neurons and nerve fibers [13]. After blocking the Kir2.1 channel, the influx of sodium ions was also inhibited, and HEK293 cell apoptosis was finally inhibited [14]. The abovementioned studies revealed that Kir2.1 played an important role in apoptosis. Previous studies have shown that the Kir2.1 channel is expressed in the microglia. However, whether it is involved in the apoptosis of BV2 and whether it is correlated with the development of hypoxia remain unclear. Therefore, the current study was aimed at investigating the effects of hypoxia on the expression of Kir2.1 protein and mRNA in BV2 and at exploring the effects of hypoxia on Kir2.1 function. In addition, the role of Kir2.1 in hypoxia-induced apoptosis of BV2 was explored preliminarily.

## 2. Materials and Methods

### 2.1. Cell Culture and Model Preparation

The microglial cell line BV2 was purchased from Procell (Wuhan, China). Fetal bovine serum (FBS, Biological Industries, the USA), 100 U/mL of penicillin (HyClone, the USA), 100 *μ*g/mL of streptomycin (HyClone, the USA), and Dulbecco's Modified Eagle Medium (containing 4500 mg/L of glucose, Gibco, the USA) were prepared into a complete medium containing 10% FBS. BV2 was cultured at 37°C and 5% CO_2_. In the experiment, BV2 cells were divided into the control, CoCl_2_ (500 *μ*mol/L, 8 h; Sigma Company, the USA), and CoCl_2_+ML133 (20 *μ*mol/L, 15 h pretreatment and coincubation with CoCl_2_ for 8 h; Sigma Company, the USA) groups.

### 2.2. CCK-8

The effect of CoCl_2_ on BV2 cell viability was detected using the CCK-8 assay. BV2 cells (8 × 10^3^/well) were inoculated with CoCl_2_ (500 *μ*M, intervention at 0, 1, 2, 4, 8, and 16 h, respectively). Then, 10 *μ*L of CCK-8 solution was added and incubated at 37°C for 1–4 h. The cell viability was determined by measuring the absorbance at 450 nm.

### 2.3. Apoptotic Cell Assay

The apoptosis rate of BV2 cells was assessed using the Annexin V-FITC assay. The cells were washed with phosphate-buffered saline (PBS) and digested with trypsin. Next, they (1 × 10^6^ cells) were suspended in 500 *μ*L of 1× binding buffer and incubated in 5 *μ*L of conjugate protein Annexin V-FITC and 10 *μ*L of PI solution at room temperature in a dark environment for 15 min. The apoptotic cells were immediately evaluated using flow cytometry, and their fluorescence at 488 nm excitation and 530 nm emission was measured. Then, the apoptotic cells were quantified using FlowJo, and the percentage of apoptotic cells was calculated.

### 2.4. Mitochondrial Membrane Potential Assay

Mitochondrial membrane potential was detected using the JC-1 Assay Kit (Abcam, the USA) following the manufacturer's instructions. BV2 cells were seeded on a 6-well culture plate and treated with CoCl_2_ and ML133. The cells were collected, and JC solution was added. The cells were washed with dilution buffer and incubated at room temperature in the dark for 20 min. The mitochondrial membrane potential was run on the FACSAria III Flow Cytometer (BD Biosciences, the USA), and the data was analyzed using FlowJo software, version 10.6.2 (Tree Star Inc., the USA).

### 2.5. Western Blot Analysis

The cells evenly spread on the six-well plate were processed, cracked, and quantified using the BCA protein quantification kit. Each sample with an equal protein content was separated via electrophoresis using 100 or 120 g/L of sodium dodecyl-sulfate polyacrylamide gel. Then, the dissolved protein was transferred to the PVDF membrane and was sealed with a sealer (containing 50 g/L of skim milk powder) at room temperature for 1.5–2 h. After sealing, a mouse anti-*β*-actin antibody (1 : 1000, Zhongshan Jinqiao Biotechnology Co., Ltd., Beijing, China), rabbit anti-Bax antibody (1 : 1000, Abcam, the USA), rabbit anti-Bcl-2 antibody (1 : 1000, Abcam, the USA), rabbit anti-caspase-3 antibody (1 : 1000, Abcam, the USA), and mouse anti-Kir2.1 antibody (1 : 1000, Abcam, the USA) were added for incubation at 4°C for at least 12 h. The PVDF membrane was cleaned with TBST and incubated with the secondary antibody for 2 h at room temperature. After washing the film again, a drop of ECL chemical developer was added to enhance the color. The gray value of the target protein was analyzed using ImageJ.

### 2.6. Real-Time Quantitative Polymerase Chain Reaction

Real-time quantitative polymerase chain reaction (PCR) was used to determine the mRNA expression of Bax, caspase-3, Bcl-2, and Kir2.1. The cells in each treatment group were collected after intervention, and the total RNA was extracted using the Trizol method. The concentration of RNA was assessed, and cDNA was obtained using the reverse transcription kit, which was then amplified using PCR. The final real-time quantitative PCR procedure was as follows: UDG enzyme activation at 50°C for 2 min, predenaturation at 95°C for 2 min, denaturation at 95°C for 15 s, and annealing/extension at 60°C for 1 min, with a total of 40 cycles. The mRNA expression levels of Bax, caspase-3, Bcl-2, and Kir2.1 were analyzed using the 2^−△△Ct^ value. All primers in the experiment were designed and synthesized by the Shanghai Shenggong Biological Company (Table 1).

### 2.7. Immunofluorescence

The protein expression and localization of Bax, caspase-3, Bcl-2, and Kir2.1 on BV2 cells were detected via immunofluorescence. BV2 cells were uniformly seeded in a 6-well plate at a density of 3 × 10^5^/mL. The slices were mounted and taken out after 24 h. After washing with PBS, the cells were fixed with 40 g/L of paraformaldehyde at room temperature for 10–20 min, and the fixative was removed. After washing with PBS, the cells in each treatment group were permeabilized with 0.2% Triton 100 for 3–5 min, and the samples were blocked with 5% BSA in a 37°C incubator for 30 min. The blocking solution was discarded. A rabbit anti-Bax antibody (1 : 100), rabbit anti-caspase-3 antibody (1 : 100), rabbit anti-Bcl-2 antibody (1 : 100), and mouse anti-Kir2.1 antibody (1 : 100) were added. The wet box was incubated overnight at 4°C and was reheated at 37°C for 30 min on the following day. A fluorescent secondary antibody (1 : 50) was added in a dark environment and incubated at 37°C for 1 h. 4′,6-Diamidino-2-phenylindole was added to stain the nucleus for 15 min in the dark, and an antifluorescence quencher was mounted. The expression and location of the target marker were assessed under a laser confocal microscope, and images were obtained. The fluorescence intensity of the target marker was analyzed using image analysis software.

### 2.8. Whole-Cell Patch-Clamp Technique

A small number of collected BV2 cells were placed in a petri dish for approximately 30 min. Meanwhile, the borosilicate glass hair embryo with core (Sutter Instrument, the USA) was drawn using the p-2000 microelectrode drawing instrument (Axon, the USA) to establish a microelectrode with a tip diameter of 1 mm and a liquid resistance of 4-8 M*Ω*. The extracellular fluid (mmol/L) was filled with NaCl 136.5, KCl 5.4, CaCl_2_ 1.8, MgCl_2_ 0.53, glucose 5.5, and HEPES 5.5 using a gravitation-driven perfusion system. NaOH was used to adjust the external liquid pH to 7.3–7.4. The electrode was injected with electrode fluid (mmol/L), which comprised K-gluconate 130, NaCl 10, CaCl_2_ 1.2, MgCl_2_ 10, HEPES 10, and EGTA 5. NaOH was utilized to adjust the liquid pH to 7.37.4. When the sealing resistance reached 1 G*Ω*, the film broke with negative pressure, thereby forming the whole-cell patch-clamp recording mode. In the voltage-clamp mode, BV2 cells were clamped at −50 mV and provided a voltage of −140–20 mV. Step stimulation with increments of 10 mV continued for 500 ms. Data were obtained using the MultiClamp 700B amplifier (Axon, the USA), filtered by 10 kHz, converted by using Digidata 1550A, a digital-to-analog/digital-to-digital converter (Axon, the USA), and analyzed using pCLAMP [15].

### 2.9. Statistical Analysis

Clampfit 10.6 was used for data analysis, and the Statistical Package for the Social Sciences 20.0 was utilized for other experimental data analyses. All experiments were repeated at least three times, and the test results were expressed as mean ± standard deviation (mean ± standard deviation). Analysis of variance was applied between the two groups. A *P* value of <0.05 was considered statistically significant. GraphPad Prism 5 was used to map the statistics.

## 3. Results

### 3.1. Hypoxia Inhibited the Activity of BV2 Cells and Induced Apoptosis

**Figure 1:**
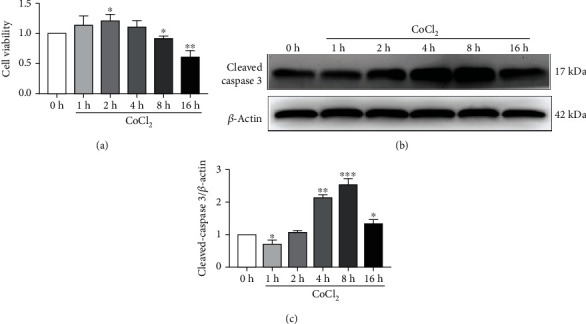
CCK-8 analysis results of cell activity changes and Western blot analysis results of cleaved caspase-3 protein expression. (a) CCK-8 detected BV2 cell activity after CoCl_2_ intervention. (b) The expression of the apoptosis protein cleaved caspase-3 in BV2 cells was assessed via Western blot analysis. (c) Semiquantitative analysis of the apoptosis protein cleaved caspase-3 levels in BV2. Mean ± standard deviation. *n* = 3. ^∗^*P* < 0.05, ^∗∗^*P* < 0.01, and ^∗∗∗^*P* < 0.001 versus the control group.

BV2 cells were treated for 0, 1, 2, 4, 8, and 16 h. As shown in Figure 1(a), the cell activity increased at 1–4 h after CoCl_2_ intervention, and the 2 h intervention group had the highest cell activity. However, that of the 8 and 16 h intervention groups decreased. Moreover, the 16 h intervention group had the lowest cell activity. However, there was no significant difference between the 1 and 4 h intervention groups and the 0 h intervention group. Meanwhile, the results significantly differed between the 8 h intervention and control groups (*P* < 0.05). The cell viability remained at >90%. The cell activity of the 16 h intervention group was significantly lower than that of the 0 h intervention group (*P* < 0.01), and its cell activity was <80%. Western blot analysis was performed to detect changes in the expression of cleaved caspase-3 after CoCl_2_ intervention at similar concentrations for different periods. As shown in Figures 1(b) and 1(c), the expression of cleaved caspase-3 increased gradually in the 0–8 h intervention groups in a time-dependent manner. Nevertheless, it decreased in the 16 h intervention group after CoCl_2_ intervention. Compared with the 0 h intervention group, there were significant differences in the 4, 8, and 16 h intervention groups, and the apoptosis of cleaved caspase-3 protein increased most significantly in the 8 h intervention group (*P* < 0.01).

### 3.2. Hypoxia Upregulated the Expression Level of Kir2.1

**Figure 2:**
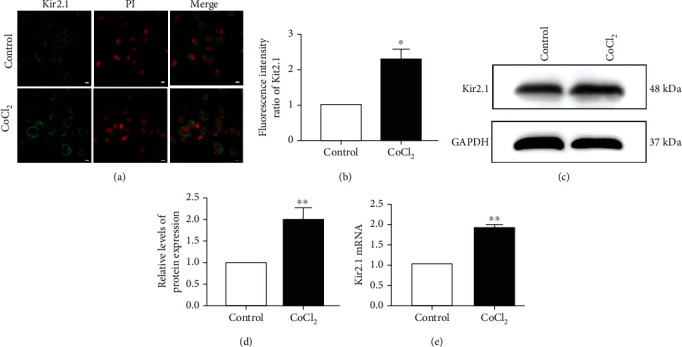
Effect of hypoxia on Kir2.1 protein expression. (a) Kir2.1 expression in BV2 (scale bar = 2.5 *μ*m). (b) Statistical analysis of the fluorescence intensity of Kir2.1 expression. (c) Kir2.1 and GAPDH blotting bands. (d) Kir2.1 protein statistical analysis diagram. (e) Kir2.1 mRNA statistical analysis diagram. Mean ± standard deviation. *n* = 3. ^∗^*P* < 0.05, ^∗∗^*P* < 0.01 versus the control group.

Cell immunofluorescence was used to detect the distribution of Kir2.1. Figures 2(a) and 2(b) show that Kir2.1 was expressed in microglia BV2, and the fluorescence expression of Kir2.1 increased in hypoxic BV2 cells (*P* < 0.05). Western blot analysis was performed to assess the effect of hypoxia on the expression of Kir2.1. Figures 2(c) and 2(d) reveal that hypoxia upregulated the expression of Kir2.1 protein in BV2 cells (*P* < 0.01). Meanwhile, PCR was conducted to detect the effect of hypoxia on Kir2.1 mRNA in BV2 cells. As shown in Figure 2(e), hypoxia upregulated Kir2.1 mRNA expression in BV2 cells (*P* < 0.01).

### 3.3. Hypoxia Increased the Inward Rectifying Potassium Channel Current I(K) in BV2 Cells

**Figure 3:**
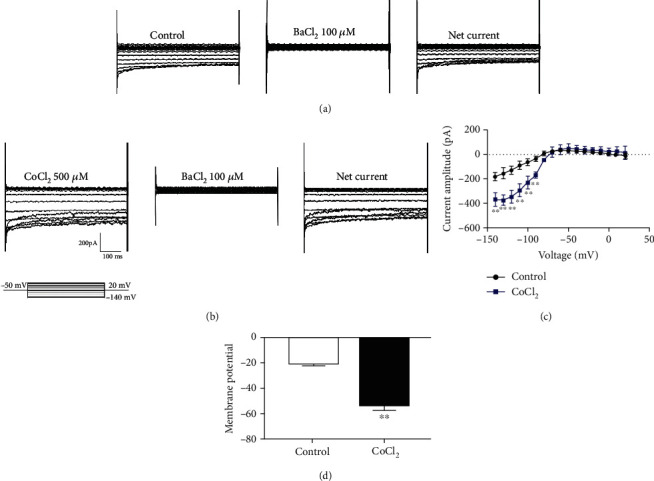
Effect of hypoxia on Kir2.1 and BV2 membrane potential. (a) Control group BV2 inward rectifier current. (b) Inward rectifying current of BV2 in the CoCl_2_ group. (c) Kir2.1 statistical diagram of the current amplitude of the net current. (d) BV2 membrane potential statistical diagram. Mean ± standard deviation. *n* = 3. ^∗^*P* < 0.05, ^∗∗^*P* < 0.01 versus the control group.

To further detect the effect of hypoxia on changes in Kir2.1 function, the whole-cell patch-clamp technique was used to detect changes in the Kir2.1 current and membrane potential in BV2 cells. As shown in Figure 3(a), the inward rectifying current could be recorded. According to the sensitivity of the inward rectifying potassium ion channel to Ba^2+^, the current could be significantly suppressed after the BV2 cells are irrigated with BaCl_2_. Moreover, it could be recorded after the cells are irrigated with extracellular fluid, thereby indicating the existence of the inward rectifying potassium current in BV2. After continuous CoCl_2_ intervention in BV2 cells for 8 h, the inward rectifier current increased, and the results significantly differed (Figure 3(b)).  Figure 3(c) shows the results of the net current in the control and CoCl_2_ groups. If the voltage ranges from −140 to −90 mV, the inward rectifier potassium current remarkably differed (*P* < 0.01).  Figure 3(d) shows the change in the membrane potential of BV2 cells. Except in the control group, hypoxia could hyperpolarize the membrane of BV2 cells.

### 3.4. ML133 Inhibited Hypoxia-Induced Apoptosis of BV2 Cells

**Figure 4:**
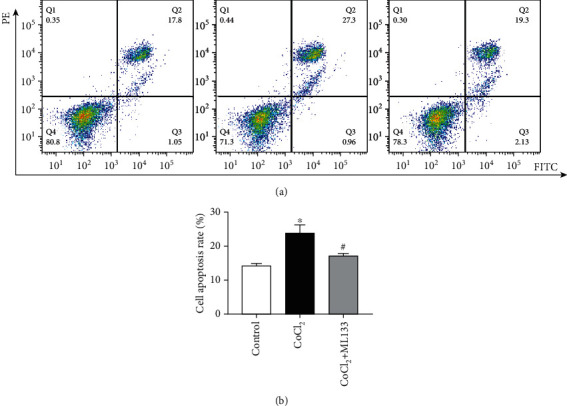
Effect of ML133 on the apoptosis of BV2 cells. (a) The apoptosis rate of BV2 cells was detected via flow cytometry. (b) Statistical diagram of the apoptosis rate of BV2 cells. Mean ± standard deviation. *n* = 3. ^∗^*P* < 0.05 versus the control group; ^#^*P* < 0.05 versus the CoCl_2_ group.

**Figure 5:**
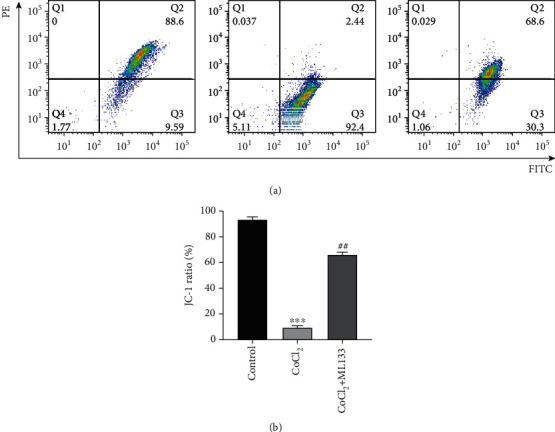
Effect of ML133 on mitochondrial membrane potential of BV2 cells. (a) The mitochondrial membrane potential of BV2 cells was detected using the JC-1 Assay Kit by flow cytometry. (b) Statistical diagram of the JC-1 ratio of BV2 cells. Mean ± standard deviation. *n* = 3. ^∗∗∗^*P* < 0.001 versus the control group; ^##^*P* < 0.01 versus the CoCl_2_ group.

**Figure 6:**
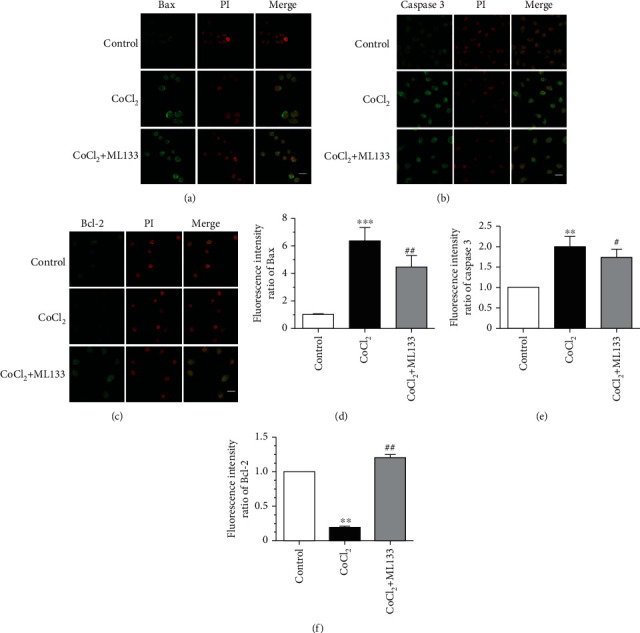
Expressions and distribution of Bax, caspase-3, and Bcl-2 on BV2 cells detected via immunofluorescence cytochemical staining. (a–c) Expression and localization of Bax, caspase-3, and Bcl-2 in BV2 (scale bar = 2.5 *μ*m). (d–f) Semiquantitative statistical analysis of fluorescence intensity. Mean ± standard deviation. *n* = 3. ^∗∗^*P* < 0.01, ^∗∗∗^*P* < 0.001 versus the control group; ^#^*P* < 0.05, ^##^*P* < 0.01 versus the CoCl_2_ group.

**Figure 7:**
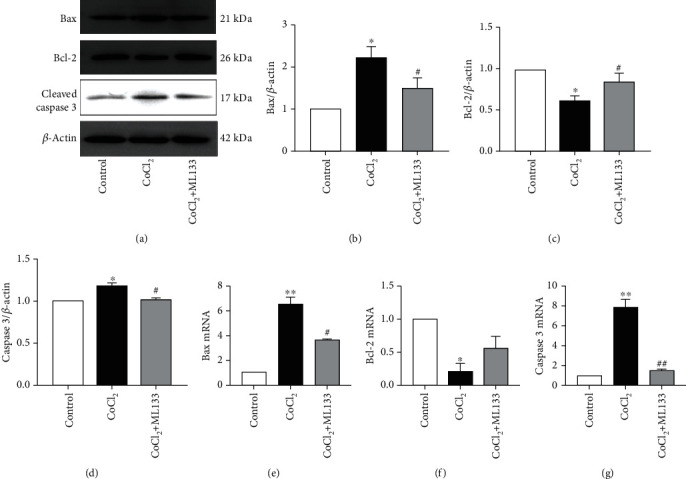
Western blot analysis and real-time quantitative polymerase chain reaction detection of Bax, caspase-3, and Bcl-2 protein expressions in BV2 cells. (a) Protein bands of Bax, Bcl-2, and cleaved caspase-3 were detected. (b–d) Bax, Bcl-2, and cleaved caspase-3 proteins were analyzed. (e–g) Bax, caspase-3, and Bcl-2 mRNA were analyzed. Mean ± standard deviation. *n* = 3. ^∗^*P* < 0.05, ^∗∗^*P* < 0.01 versus the control group; ^#^*P* < 0.05, ^##^*P* < 0.01 versus the CoCl_2_ group.

Flow cytometry was used to detect the rate of apoptosis in BV2 cells. As shown in Figures 4(a) and 4(b), the apoptosis rate of the CoCl_2_ intervention group significantly increased compared with that of the control group (*P* < 0.05). Meanwhile, the apoptosis rate of the CoCl_2_+ML133 intervention group significantly decreased compared with that of the CoCl_2_ intervention group (*P* < 0.05). The expression of apoptosis proteins caspase-3 and Bax and the antiapoptosis protein Bcl-2 was detected using immunofluorescence. As shown in Figure 5, CoCl_2_ significantly increased mitochondrial membrane potential, compared to untreated BV2 cells. ML133 attenuated mitochondrial membrane potential compared with the CoCl_2_ group (*P* < 0.05). The expression of apoptosis proteins caspase-3 and Bax and the antiapoptosis protein Bcl-2 was detected by immunofluorescence. As shown in Figure 6, the expression of Bax and caspase-3 increased in the treatment groups, and the expression of Bcl-2 decreased in the CoCl_2_ intervention group. Compared with the CoCl_2_ intervention group, the expression of caspase-3 and Bax decreased in the CoCl_2_+ML133 intervention group. Meanwhile, the expression of Bcl-2 increased (*P* < 0.05). To further validate the effect of ML133 on the apoptosis of BV2 cells, Western blot analysis was performed to detect the expression of apoptosis proteins caspase-3 and Bax and the antiapoptosis protein Bcl-2. As shown in Figures 7(a)–7(d), the expressions of cleaved caspase-3 and Bax increased and that of Bcl-2 decreased in the CoCl_2_ intervention group. The expression of cleaved caspase-3 and Bax decreased, and that of Bcl-2 increased in the CoCl_2_+ML133 intervention group (*P* < 0.05). As depicted in Figures 7(e)–7(g), the expressions of cleaved caspase-3 and Bax increased and the expression of Bcl-2 decreased in the CoCl_2_ intervention group. The expressions of cleaved caspase-3 and Bax decreased, and the expression of Bcl-2 increased in the CoCl_2_+ML133 intervention group (*P* < 0.05).

## 4. Discussion

This study was aimed at investigating the role and mechanism of Kir2.1 in microglia apoptosis induced by hypoxia. In this research, CoCl_2_, a chemical hypoxic agent, was used to prepare the BV2 microglial cell line hypoxic model and to explore the role of Kir2.1 in the apoptosis of BV2 cells induced by hypoxia. Results showed that hypoxia not only upregulated the protein expression of Kir2.1 but also enhanced the function of the Kir2.1 channel. After the administration of ML133, a Kir2.1 channel blocker, the apoptosis of hypoxia-induced BV2 cells was inhibited. Thus, Kir2.1 may be involved in the apoptosis process of hypoxia-induced BV2 cells.

CoCl_2_ is the most commonly used chemical reagent in the preparation of hypoxic/ischemic cell models, and the mitochondrial membrane potential and ROS of cells can decrease after CoCl_2_ intervention. The production of ROS [7] increases, further triggering the hypoxic state of cells, which then affects their normal physiological functions [16]. Previous studies have shown that CoCl_2_ can induce apoptosis of different cells, and CoCl_2_ can induce cellular dysfunction of human periodontal membrane stem cells (PDLSCs) in a concentration-dependent manner, leading to the excessive production and accumulation of ROS in PDLSCs and inducing apoptosis via the ROS-dependent pathway [17]. In this study, the CCK-8 analysis showed that the cell activity remained >90% after CoCl_2_ intervention for 8 h, thereby indicating good cell activity. The 16 h intervention had a significant effect on cell viability, which was not conducive to cell survival and subsequent experiments. Western blot analysis results showed that changes in the expression of cleaved caspase-3 were most significant after CoCl_2_ treatment for 8 h. Therefore, 500 *μ*mol/L of CoCl_2_ was used to intervene with BV2 cells for 8 h to prepare the BV2 apoptosis model.

TN is an intense, stabbing, electric shock-like pain caused by irritation of the trigeminal nerve and is occasionally described as the most excruciating pain known among humans. TN has several causes. Among them, maxillofacial pathology has been increasingly recognized [18]. Previous studies have shown that hypoxia can induce oxidative stress in cells [19]. Other reports have revealed that oxidative stress byproducts are a key factor in the development and maintenance of trigeminal pain, which provides a new explanation for the pathophysiology of trigeminal neuropathic pain [10]. Microglia are innate immune cells that play a key regulatory role in immune response to oxidative stress.

KIRs are named for their unique voltage-current relationship, which is characterized by their inward rectification properties. Kir comprises seven subfamilies (Kir1.x-Kir7.x), each of which has its own functional characteristics. For example, Kir2.x is expressed in excitable cells (cardiomyocytes, skeletal muscle cells, and nerve cells). The overexpression of the Kir2.1 channel protein in excitatory neurons inhibits excitatory neuron activity. The inhibition of excitatory neuronal activity can aggravate the injury of subacute ischemic stroke in mice, promote neuronal death, and delay the recovery of neurological function after ischemic stroke in mice. Previous studies have shown that Kir2.1 is expressed in both primary microglia and BV2 cells, and it can affect the cell migration process via the function of potassium channels [20]. In addition, potassium ion channels can stabilize and regulate the membrane potential of glial cells cultured in vitro [21]. This study confirmed that hypoxia upregulated the expression of Kir2.1 and enhanced its channel function in BV2 cells.

The mitochondrial apoptosis pathway is one of the three classical apoptosis pathways, and blocking the apoptosis pathway can effectively reduce TN caused by oxidative stress [22]. CoCl_2_ can downregulate mitochondrial membrane potential. The integrity of mitochondrial membrane potential depends on the antagonism between antiapoptotic and apoptotic proteins in cells [23]. CoCl_2_ can inhibit the activity of BV2 cells and induce apoptosis. After ML133 was administered, the expressions of proapoptotic proteins Bax and cleaved caspase-3 decreased. Meanwhile, the expressions of the antiapoptotic protein Bcl-2 increased. Thus, ML133 can inhibit the hypoxia-induced apoptosis of BV2 cells.

## 5. Conclusion

Kir2.1 is involved in the hypoxia-induced apoptosis of BV2 cells. Microglia play an important role in the nervous system. Thus, the inhibition of microglia apoptosis can improve hypoxic injury in the nervous system, thereby inhibiting the development of TN. However, whether it plays a role in combination with the signal pathway must be further evaluated. It is believed that with the further development of research, the biological role and regulatory mechanism of microglia in TN will be further improved and provide new ideas and targets for the diagnosis, treatment, and prognosis of TN.

## Figures and Tables

**Table 1:** Sequences of the primers.

Name	Primer sequence	Product (bp)
Bax	Forward: 5′-CCAGGACGCATCCACCAAGAAG-3′	138
	Reverse: 5′-GCTGCCACACGGAAGAAGACC-3′	

Caspase-3	Forward: 5′-GTACAGAGCTGGACTGCGGTATTG-3′	84
	Reverse: 5′-AGTCGGCCTCCACTGGTATCTTC-3′	

Bcl-2	Forward: 5′-ACGGTGGTGGAGGAACTCTTCAG-3′	168
	Reverse: 5′-GGTGTGCAGATGCCGGTTCAG-3′	

Kir2.1	Forward: 5′-ATGGGCAGTGTGAGAACCAAC-3′	113
	Reverse: 5′-TGGACTTTACTCTTGCCATTCC-3′	

## Data Availability

The data that support the findings of this study are available from the corresponding author upon reasonable request.
